# Anticonvulsant Activity of Extracts of *Plectranthus barbatus* Leaves in Mice

**DOI:** 10.1155/2012/860153

**Published:** 2011-06-09

**Authors:** Luciana Cristina Borges Fernandes, Carlos Campos Câmara, Benito Soto-Blanco

**Affiliations:** Departamento de Ciências Animais, Universidade Federal Rural do Semi-Árido (UFERSA), BR 110 Km 47, 59625-900 Mossoró, RN, Brazil

## Abstract

*Plectranthus barbatus* is a medicinal plant used to treat a wide range of disorders including seizure. However, the anticonvulsant activity of this plant has not been studied in depth. We therefore sought to evaluate the anticonvulsant activity of a hydroalcoholic extract of *P. barbatus* leaves on seizures induced by strychnine sulphate (2.0 mg/kg) and pilocarpine (600 mg/kg) in mice. The extract was administered orally at 1, 10, 30, and 100 mg/kg. We report that the *P. barbatus* extract had marked anticonvulsant activity against strychnine-induced convulsions, but was quite ineffective against pilocarpine-induced convulsions. Further experiments will be required to identify the active molecules(s) and their mechanism(s) of action.

## 1. Introduction

*Plectranthus barbatus* Andr. is a perennial shrub that is thought to have originated in Africa and is used as a medicinal plant to treat a wide range of disorders. The plant is also known by the synonyms *Plectranthus forskohlii* Briq, *Plectranthus forskalaei* Willd., *Plectranthus kilimandschari* (Gürke) H.L. Maass., *Plectranthus grandis* (Cramer) R.H. Willemse, *Coleus forskohlii* Briq., *Coleus kilimandschari* Gürke ex Engl., *Coleus coerulescens* Gürke, and *Coleus barbatus* (Andr.) Benth [1]. The medicinal uses of *P. barbatus* include digestive, respiratory, circulatory, nervous disorders [1] and infections [2] and has been reported to have abortifacient, contraceptive [3], and antipain [4] properties. The plant is also used to treat gastritis and intestinal spasms [5], nausea [6], stomach ache, and as a purgative [7]. Uses in respiratory disorders include the relief of colds [8], cough, [4] and bronchitis [9], and in the circulatory system uses include myalgia, angina, and hypertension [4].

*P. barbatus* is also used for the treatment of epilepsy and convulsions [10]; however, only limited data are available concerning the anticonvulsant activity of this plant. The present work was undertaken to evaluate the anticonvulsant activity of an extract of *Plectranthus barbatus* leaves.

## 2. Methods

### 2.1. Plant Extract Preparation

Fresh leaves of cultivated *Plectranthus barbatus* were obtained from the Medicinal and Toxic Plants Garden, Sector of Seedlings Production, Universidade Federal Rural do Semi-Árido—UFERSA, Mossoró, RN, Brazil. A reference specimen is deposited in the Prisco Bezerra Herbarium at the Universidade Federal do Ceará, Fortaleza, CE, Brazil, under number 24408. Leaves were air-dried at 35°C, pulverized, and the powder was extracted with ethanol : water (20 : 80), filtered, and dried by evaporation. The dry extract was dissolved in distillated water at concentration of 50 mg dried *P. barbatus* solids per ml.

### 2.2. Animals

Swiss albino male mice, weighing between 25 to 30 g, two months old, were kept under controlled conditions (21–25°C on a 12 h : 12 h light : dark cycle). Animals were maintained on a standard mouse diet; before the experiments they were fasted overnight with water *ad libitum*.

Ethical procedures were based on the Brazilian law 6638 (May 8, 1979) “Normas para Prática Didático-Científica da Vivissecção de Animais” and “Ethical Principles for Use of Experimental Animals” from Colégio Brasileiro de Experimentação Animal (COBEA), Brazil, which are in accordance with the “European Convention for the Protection of Vertebrate Animals used for Experimental and Other Scientific Purposes” (Strasbourg, March 18, 1986).

### 2.3. Evaluation of Anticonvulsant Activity

For each seizure-inducing drug tested, 40 mice were divided into five groups of eight animals; *P. barbatus* extract was administered by oral gavage at doses of 0, 1, 10, 30, and 100 mg/kg. Thirty minutes after administration, each mouse was injected intraperitoneally with strychnine (2.0 mg/kg) or with pilocarpine (600 mg/kg). The doses of used drugs were defined in a pilot study. The latency to first convulsion and the latency and percentage mortality were recorded for a period of 30 min. Animals surviving more than 30 min were considered to be protected.

### 2.4. Statistical Analysis

Results are expressed as means ± standard error of the mean (SEM). Comparisons between the averages of series of values were performed by ANOVA followed by Dunnett's multiple comparisons test. Data analysis employed Graphpad INSTAT version 2.0 software; statistical significance was set at *P* < .05.

## 3. Results

*P. barbatus* extract at doses of 1 and 100 mg/kg significantly (*P* < .05) delayed the onset of strychnine-induced seizures (Table 1). At a dose of 1 mg/kg, there was a significant (*P* < .05) increase in the latency to death, but the extract failed to protect the animals against drug-induced death. At a dose of 10 mg/kg, there was no significant difference in time before onset of seizure or death latency. However, *P. barbatus* extract at dose of 30 mg/kg protected 12.5% (1/8) of treated animals; although time to seizure onset and latency to death were increased by extract administration the difference failed to achieve statistical significance (*P* > .05). At 100 mg/kg, all treated animals (8/8) were now protected against drug-induced death (*P* < .0001).

For pilocarpine-induced seizures, all *P. barbatus* extract doses (1, 10, 30, and 100 mg/kg) significantly (*P* < .05) delayed seizure onset, and doses of 1, 10, and 100 mg/kg significantly (*P* < .05) increased the latency to death. However, no significant retardation of death latency at a dose of 30 mg/kg was seen compared to the control group, and no dose protected animals from convulsions or death (Table 2).

## 4. Discussion

We report that a hydroalcoholic extract of leaves of *Plectranthus barbatus* exerts anticonvulsant effects against both strychnine- and pilocarpine-induced seizures. All tested doses of *P. barbatus* extract retarded pilocarpine-induced seizures but failed to protect against death. By contrast, no seizure activity and no death were observed in strychnine-treated animals pretreated with the highest tested dose of *P. barbatus* extract (100 mg/kg).

The mechanism underlying strychnine-induced seizures is thought to involve direct antagonism of strychnine-sensitive glycine receptors not only in higher brain areas but also in the spinal cord and brainstem, thereby abrogating spinal reflexes and causing motor disturbance, increased muscle tone, hyperactivity of sensory, visual and acoustic perception, tonic convulsions, and death through respiratory or spinal paralysis or by cardiac arrest [11]. Our results demonstrate that strychnine-induced seizures are partly suppressed by *P. barbatus *treatment.

Pilocarpine is a nonspecific muscarinic acetylcholine receptor agonist; it has been suggested that cerebral structures with a high density of muscarinic receptors are likely to represent the sites of origin of acute pilocarpine seizures [12]. Pilocarpine administration to rodents leads to repetitive limbic seizures and status epilepticus, replicating several features of human temporal lobe epilepsy [13]. It was previously suggested that extracts of *P. amboinicus* might antagonize cholinergic action at muscarinic receptors [14]. On the other hand, *P. barbatus* extracts were previously reported to inhibit acetylcholinesterase activity [15]. In the present work, the administration of *P. barbatus* extracts failed to inhibit pilocarpine-induced seizures, even though it retarded the onset of the seizures. Thus, it appears likely that *P. barbatus* extract presented slight antagonist action in the muscarinic acetylcholine receptors. 

Phytochemical studies on *P. barbatus* have revealed numerous bioactive compounds: notably diterpenoids including forskolin [16], plectranthone J, plectrin, (16S)-coleon E, coleon F, (16R)-plectrinone A, plectrinone B [17], cyclobarbatusin, barbatusin, and 7*β*-acetyl-12-desacetoxicyclobarbatusin [18]. Of these, forskolin is a promising candidate for the anticonvulsant activity of *P. barbatus* because this molecule was shown to suppress seizures induced by pentylenetetrazol [19]. Forskolin activates adenylate cyclase and increases intracellular cAMP levels. It was previously reported that forskolin can elicit membrane depolarization in medium spiny neurons of the mouse nucleus accumbens; this was mediated by cAMP stimulation of inward rectifier K^+^ currents [20]. Forskolin increases the amplitude of glycinergic miniature inhibitory postsynaptic currents in the presence of low concentrations of extracellular glycine in isolated rat substantia gelatinosa neurons [21].

In addition to direct effects on ion channels, forskolin treatment is likely to exert neuroprotective effects by increasing cellular levels of neurotrophin-3 (NT-3) [22], a member of the neurotrophin family of neurotrophic factors that support cell survival, axonal growth, and neuronal plasticity [23, 24]. Brain injury decreases NT-3 synthesis in the hippocampus [25], whereas NT-3 supplementation can protect neurons against excitotoxic insult [26]. Seizures induced by kainic acid [27] or pilocarpine [28] injection decreased NT-3 expression in the brain of mice, whereas intraventricular infusion of NT-3 in rats inhibited kindling epileptogenesis, mossy fiber sprouting, and increases in hilar area [29], arguing that NT-3 upregulation by forskolin is likely to inhibit seizure development and seizure-related synaptic reorganization.

## 5. Conclusion

In conclusion, *P. barbatus* extract was found to have marked anticonvulsant activity against strychnine-induced convulsions, but was quite ineffective against pilocarpine-induced convulsions. Although forskolin is a promising candidate molecule for the anticonvulsant activity of *P. barbatus* extracts, it is possible that the bioactivity is mediated by a combination of two or more molecules. Further experiments will be required to identify the active molecules(s) and their mechanism(s) of action.

## Figures and Tables

**Table 1:** Anticonvulsant effect of *Plecthranthus barbatus* leaf extracts on strychnine-induced seizures in mice.

Dose (mg/kg) p.o.	Time to seizure onset (s)	Death latency (s)	Death rate (%)
0 (control)	217 ± 23.9	282.0 ± 46.7	100
1	461.3 ± 68.1*	518.8 ± 48.6*	100
10	236.4 ± 51.3	353.0 ± 46.7	100
30	317.4 ± 45.4	452.3 ± 52.6	87.5
100	no seizures	no seizures	0

*P < .05, compared to control (ANOVA followed by Dunnett's multiple comparisons test).

**Table 2:** Anticonvulsant effects of *Plecthranthus barbatus* extract on pilocarpine-induced seizures in mice.

Dose (mg/kg) p.o.	Time to seizure onset (s)	Death latency (s)	Death rate (%)
0 (control)	117.1 ± 14.8	194.7 ± 19.9	100
1	300.9 ± 53.3*	364.5 ± 59.4*	100
10	373.9 ± 18.7*	468.1 ± 39.6*	100
30	276.4 ± 20.6*	317.1 ± 26.9	100
100	261.0 ± 29.1*	334.0 ± 25.2*	100

*P < .05, significant difference compared to control (ANOVA followed by Dunnett's multiple comparisons test).

## References

[1] Lukhoba CW, Simmonds MSJ, Paton AJ (2006). *Plectranthus*: a review of ethnobotanical uses. *Journal of Ethnopharmacology*.

[2] Gupta S, Yadava JNS, Tandon JS (1993). Antisecretory (antidiarrhoeal) activity of Indian medicinal plants against *Escherichia coli* enterotoxin-induced secretion in rabbit and guinea pig ileal loop models. *International Journal of Pharmacognosy*.

[3] Almeida FCG, Lemonica IP (2000). The toxic effects of *Coleus barbatus* B. on the different periods of pregnancy in rats. *Journal of Ethnopharmacology*.

[4] Chifundera K (2001). Contribution to the inventory of medicinal plants from the Bushi area, South Kivu Province, Democratic Republic of Congo. *Fitoterapia*.

[5] Câmara CC, Nascimento NRF, Macêdo-Filho CL, Almeida FBS, Fonteles MC (2003). Antispasmodic effect of the essential oil of *Plectranthus barbatus* and some major constituents on the guinea-pig ileum. *Planta Medica*.

[6] Hamill FA, Apio S, Mubiru NK (2003). Traditional herbal drugs of Southern Uganda—II: literature analysis and antimicrobial assays. *Journal of Ethnopharmacology*.

[7] Johns T, Kokwaro JO, Kimanani EK (1990). Herbal remedies of the Luo of Siaya District, Kenya: quantitative criteria for consensus. *Economic Botany*.

[8] Rajendran A, Rao NR, Ravikumar K, Henry AN (1999). Some medicinal and aromatic labiates from the Peninsular India. *Journal of Non-Timber Forest Products*.

[9] Cos P, Hermans N, De Bruyne T (2002). Further evaluation of Rwandan medicinal plant extracts for their antimicrobial and antiviral activities. *Journal of Ethnopharmacology*.

[10] Schaneberg BT, Khan IA (2003). Quantitative analysis of forskolin in *Coleus forskohlii* (Lamiaceae) by reversed-phase liquid chromatography. *Journal of AOAC International*.

[11] Philippe G, Angenot L, Tits M, Frédérich M (2004). About the toxicity of some *Strychnos* species and their alkaloids. *Toxicon*.

[12] Clifford DB, Olney JW, Maniotis A, Collins RC, Zorumski CF (1987). The functional anatomy and pathology of lithium-pilocarpine and high-dose pilocarpine seizures. *Neuroscience*.

[13] Quintans LJ, Almeida JRGS, Lima JT (2008). Plants with anticonvulsant properties—a review. *Brazilian Journal of Pharmacognosy*.

[14] Pérez-Saad H, Buznego MT, Llanio M, Fernández MD, Menéndez R (2003). Neuropharmacological profile of *Plectranthus amboinicus* (Lour.) Spreng. *Revista de Neurologia*.

[15] Falé PL, Borges C, Madeira PJA (2009). Rosmarinic acid, scutellarein 4′-methyl ether 7-O-glucuronide and (16S)-coleon E are the main compounds responsible for the antiacetylcholinesterase and antioxidant activity in herbal tea of *Plectranthus* barbatus (“falso boldo”). *Food Chemistry*.

[16] ValdÉs LJ, Mislankar SG, Paul AG (1987). *Coleus barbatus* (*C. forskohlii*)(Lamiaceae) and the potential new drug forskolin (Coleonol). *Economic Botany*.

[17] Abdel-Mogib M, Albar HA, Batterjee SM (2002). Chemistry of the genus *Plectranthus*. *Molecules*.

[18] De Albuquerque RL, Kentopff MR, Machado MIL (2007). Abietane diterpenoids isolation from *Plectranthus* barbatus. *Quimica Nova*.

[19] Sano M, Seto-Ohshima A, Mizutani A (1984). Forskolin supresses seizures induced by pentylenetetrazol in mice. *Experientia*.

[20] Podda MV, Riccardi E, D’Ascenzo M, Azzena GB, Grassi C (2010). Dopamine D1-like receptor activation depolarizes medium spiny neurons of the mouse nucleus accumbens by inhibiting inwardly rectifying K^+^ currents through a cAMP-dependent protein kinase A-independent mechanism. *Neuroscience*.

[21] Nakamura M, Jang ILS (2010). GlyT-2 mediates the forskolin-induced increase of glycinergic transmission. *NeuroReport*.

[22] Mele T, Čarman-Kržan M, Jurič DM (2010). Regulatory role of monoamine neurotransmitters in astrocytic NT-3 synthesis. *International Journal of Developmental Neuroscience*.

[23] Maisonpierre PC, Belluscio L, Squinto S (1990). Neurotrophin-3: a neurotrophic factor related to NGF and BDNF. *Science*.

[24] Davies AM (2000). Neurotrophins: neurotrophic modulation of neurite growth. *Current Biology*.

[25] Hicks RR, Martin VB, Zhang L, Seroogy KB (1999). Mild experimental brain injury differentially alters the expression of neurotrophin and neurotrophin receptor mRNAs in the hippocampus. *Experimental Neurology*.

[26] Cheng B, Mattson MP (1994). NT-3 and BDNF protect CNS neurons against metabolic/excitotoxic insults. *Brain Research*.

[27] Lee S, Williamson J, Lothman EW (1997). Early induction of mRNA for calbindin-D(28k) and BDNF but not NT-3 in rat hippocampus after kainic acid treatment. *Molecular Brain Research*.

[28] Hagihara H, Hara M, Tsunekawa K, Nakagawa Y, Sawada M, Nakano K (2005). Tonic-clonic seizures induce division of neuronal progenitor cells with concomitant changes in expression of neurotrophic factors in the brain of pilocarpine-treated mice. *Molecular Brain Research*.

[29] Xu B, Michalski B, Racine RJ, Fahnestock M (2002). Continuous infusion of neurotrophin-3 triggers sprouting, decreases the levels of TrkA and TrkC, and inhibits epileptogenesis and activity-dependent axonal growth in adult rats. *Neuroscience*.

